# Color Recurrence Plots from Uniform Delay Embeddings for Bearing Degradation Tracking and Prognostics

**DOI:** 10.3390/e28060668

**Published:** 2026-06-11

**Authors:** Algirdas Kazlauskas, Rita Baublienė, Mantas Landauskas, Minvydas Ragulskis

**Affiliations:** Department of Mathematical Modelling, Kaunas University of Technology, Studentu 50, LT 51368 Kaunas, Lithuania; algirdas.kazlauskas@ktu.edu (A.K.); rita.palivonaite@ktu.lt (R.B.); mantas.landauskas@ktu.lt (M.L.)

**Keywords:** phase-space reconstruction, time-delay embedding, uniform delay vector, non-uniform delay vector, recurrence plot, color recurrence plot, bearing vibration, remaining useful life prediction

## Abstract

Prognostic health management of rolling element bearings requires feature representations that reliably track degradation while remaining tractable for real-time deployment. This paper investigates whether uniform time-delay embedding can serve as a near-optimal substitute for computationally expensive non-uniform embedding in recurrence-based vibration analysis. We show empirically that optimally chosen uniform delay vectors yield phase-space reconstructions of bearing vibration signals not significantly inferior to those produced by globally optimized non-uniform delay vectors, compressing the parameter search from a combinatorial optimization to a single scalar selection. Building on this near-optimality result, we construct color recurrence plots from uniformly embedded phase spaces and apply them to remaining useful life (RUL) prediction on the Intelligent Maintenance Systems (IMS) bearing dataset. We further demonstrate that standard binary recurrence plots are poorly suited for RUL estimation: their dense and erratically varying local patterns obscure the degradation trends required for reliable prognostics. Color recurrence plots, by contrast, suppress these local instabilities by averaging recurrence structures across multiple phase-space projections, exposing a globally evolving intensity that tracks bearing health throughout its degradation trajectory. This work establishes uniform delay embedding combined with color recurrence representation as an efficient, principled, and practically deployable approach to recurrence-based condition monitoring in industrial predictive maintenance.

## 1. Introduction

Rolling element bearings are among the most degradable components in rotating machinery. Their progressive degradation directly affects system reliability, operational safety, and maintenance costs across a wide range of industrial applications. Bearing faults display primarily as characteristic disturbances in vibration responses, making vibration-based condition monitoring the predominant approach for fault diagnosis and remaining useful life (RUL) prediction [1,2]. Benchmark run-to-failure datasets, such as the IMS bearing dataset from the NASA Ames Prognostics Data Repository, have been instrumental in the development and validation of these methods [3,4].

Raw vibration signals from operating bearings are typically nonlinear, chaotic, and usually contaminated by noise. In early degradation stages, fault-related signatures are weak and often indistinguishable from normal variability; more distinctive patterns emerge only after the system transitions into an accelerated degradation regime [5,6]. This two-phase behavior, consisting of a relatively stable period followed by rapid deterioration, motivates the development of signal representations that capture nonlinear temporal structure, enable reliable detection of degradation-onset transitions, and provide informative features for prognostic models. Entropy-based measures have gained traction in this context, as they quantify the complexity and irregularity of vibration signals in ways that correlate with bearing health [7,8]. However, scalar feature extraction does not fully capture the geometric structure of the underlying dynamical system, which motivates the use of phase-space reconstruction as a complementary approach.

Phase-space reconstruction is a method for extracting nonlinear dynamical information from measured time series. Embedding theory demonstrates that the latent dynamics of a smooth deterministic system can be reconstructed from time-delayed observations of a single scalar observable, provided appropriate conditions on the embedding dimension and time delay are met [9,10]. In standard uniform delay embedding, the reconstructed state vector is defined as $y_{t}=\left[x_{t},\;x_{t+\tau },\;\ldots ,\;x_{t+(m-1)\tau }\right]$, where *m* is the embedding dimension and τ is the uniform time delay. This approach is advantageous because it reduces parameter selection to two scalars. Non-uniform embedding extends this method by assigning distinct delays to each coordinate, offering greater flexibility when the observed dynamics involve multiple time scales [11,12]. However, this flexibility comes at the cost of a substantially enlarged parameter space, which poses practical challenges when embedding serves as a preprocessing step in the prognostic pipeline.

Selecting proper embedding parameters is a critical prerequisite, as these choices govern the geometry of the reconstructed trajectory and can strongly influence the quality of subsequent classification, forecasting, or prognostic analyses. Classical heuristics handle this by selecting the time delay as either the first zero of the autocorrelation function or the first minimum of the average mutual information (AMI), while the embedding dimension is commonly estimated by the false nearest neighbors (FNNs) criterion [13,14]. These criteria have found broad adoption because they are relatively inexpensive to compute and yield readily interpretable results. Phase-space reconstruction based on these heuristics has been applied throughout diverse domains. In power systems, Cai et al. used AMI-based delay selection to convert one-dimensional power-quality disturbance signals into phase-space images for convolutional neural network (CNN) classification [15]. In structural health monitoring, Chen et al. employed high-dimensional phase-space reconstruction, similarly guided by mutual information and autocorrelation criteria, to generate attractor image representations for CNN-based damage detection [16]. These studies illustrate how reconstructed state-space representations may serve as analytical tools for nonlinear dynamics characterization and as image-like inputs for modern deep learning pipelines.

Despite their practical utility, heuristic delay-selection rules can become insufficient when the underlying dynamics are multiscale, intermittent, or only partially observable. This limitation has prompted increased research into non-uniform and automated embedding strategies. Han et al. demonstrated that selective non-uniform delay-coordinate assignment improves forecast precision for multivariate chaotic time series by focusing the embedding on the most informative lags [17]. Gao et al. proposed a non-uniform delay-coordinate multiscale predictor for blast furnace systems, demonstrating that heterogeneous lag selection yields measurable gains in prediction performance for complex industrial processes [18]. In the biomedical domain, Gu and Chou applied non-uniform multivariate embedding of electroencephalography (EEG) signals to detect epileptic seizure onset, leveraging the sensitivity of non-uniform embeddings to abrupt dynamical transitions [19]. The connection to bearing prognostics is direct: the onset of accelerated bearing degradation represents a qualitatively similar dynamical transition, from a slowly evolving steady state to a rapidly deteriorating regime.

More recent work has reframed embedding parameter selection as an explicit optimization problem. Kraemer et al. developed a unified and automated method for attractor reconstruction that combines non-uniform delay selection with objective-function-driven embedding dimension estimation, eliminating reliance upon ad hoc heuristics [20]. In a complementary study, the same group introduced a Monte Carlo decision tree search strategy for optimal state-space reconstruction, enabling efficient exploration of the embedding space when exhaustive enumeration of candidate delay vectors is computationally prohibitive [21]. Krakovská et al. conducted a systematic comparison of reconstruction techniques—including uniform, non-uniform, weighted-delay, principal-component, and differential approaches—and found that predictive accuracy is strongly dependent on the chosen reconstruction strategy, with no single method universally superior across tasks [22]. Embedding delays can also be selected using topological criteria; Tan et al. proposed a persistent homology-based approach that provides a geometrically principled alternative to mutual information or autocorrelation-based heuristics [23]. Together, these findings support the view that optimal embedding should be defined relative to the specific task, rather than by a universal geometric or information-theoretic criterion.

Recurrence analysis offers a natural and powerful framework for converting reconstructed phase-space trajectories into structured visual and quantitative representations. Recurrence plots, introduced by Eckmann et al., visualize the tendency of a dynamical trajectory to revisit the same region of state space, encoding the full recurrence structure as a symmetric binary matrix [24]. Since their introduction, recurrence plots and the associated recurrence quantification analysis (RQA) have become widely used tools for analyzing nonlinear and non-stationary systems, with applications in geophysics, physiology, engineering, and finance [25]. Recurrence-based measures, including determinism, entropy, laminarity, trapping time, and diagonal-line length statistics, have proven sensitive to changes in dynamical behavior that accompany structural faults and system degradation. Kecik et al. demonstrated this sensitivity explicitly in the context of ball bearing fault diagnosis, showing that RQA indicators respond reliably to fault-induced changes in bearing dynamics [26]. Building on these results, Petrauskiene et al. introduced color recurrence plots for bearing fault diagnosis by converting embedded vibration trajectories into three-channel color image representations suitable for image-based classification pipelines [27]. Related work has further explored the utility of phase-space representations for structural damage detection using Koopman operator methods [28] and for seizure classification using recurrence-derived features from EEG signals [29]. Collectively, these studies demonstrate a methodology that links phase-space reconstruction, recurrence image generation, and condition monitoring.

Despite these advances, the majority of recurrence-based bearing studies have focused on fault detection or fault-type classification, with comparatively limited attention to RUL prediction. Existing prognostic research has explored degradation modeling [30], entropy-based feature extraction [31], and fault identification under variable operating conditions [32]. While these works demonstrate that nonlinear representations can facilitate bearing prognosis, none directly addresses the selection of a uniform delay embedding suited to color recurrence plot construction, where the primary objective is to detect the onset of accelerated degradation and to predict RUL from that point onward.

Although non-uniform embedding provides considerable modeling flexibility, it simultaneously increases the complexity and computational cost of parameter selection. In high-dimensional phase spaces, the number of candidate delay-coordinate combinations grows rapidly, rendering exhaustive search unsuitable for real-time deployment. Constructing color recurrence plots in this context may require evaluating many delay vectors before a suitable reconstruction is identified [27]. A uniform delay vector offers a simpler alternative: it collapses the combinatorial search to a single scalar, preserves interpretability, and is consistent with established embedding heuristics.

The present study considers this problem by examining the selection of an optimal uniform delay embedding for bearing vibration time series. The selected uniform delay vector is used to construct color recurrence plots, which are then applied to RUL prediction on the IMS bearing dataset. Optimality is defined exclusively in terms of downstream prognostic utility: a uniform delay embedding is preferred if the resulting recurrence representations more clearly reveal the transition to accelerated deterioration, and yield improved RUL prediction accuracy after that transition. This framework provides a computationally efficient, practically deployable approach to recurrence-based condition monitoring for industrial predictive maintenance.

## 2. Materials and Methods

### 2.1. The Description of the Dataset

The experimental dataset used in this research was sourced from the University of Cincinnati’s Center for Intelligent Maintenance Systems (IMS) via the NASA Prognostics Data Repository [3]. The IMS bearing dataset is publicly available and has been widely used as a benchmark for fault diagnosis and prognostic studies [25]. It consists of three run-to-failure experiments conducted on a test rig with four Rexnord ZA-2115 double-row spherical roller bearings mounted on a common rotating shaft, driven at a constant speed of 2000 RPM under a radial load of 6000 lbs. Vibration signals were recorded by PCB 353B33 high-sensitivity quartz ICP accelerometers mounted on the bearing housing. Each recorded file contains 20,480 samples acquired at a sampling frequency of 20 kHz, yielding one second of raw vibration data per file. The data were collected at 10 min intervals throughout each run-to-failure experiment, except for the first 43 files in Dataset 1, which were recorded every 5 min to capture the early stages of degradation.

Table 1 provides an overview of the dataset structure, including the three subsets with varying numbers of files and accelerometer channels. Bearing faults were introduced and observed near the conclusion of the experiments: Dataset 1 includes an inner-race fault in Bearing 3 and a rolling-element defect in Bearing 4; Dataset 2 contains an outer-race fault in Bearing 1; and Dataset 3 contains an outer-race fault in Bearing 3. In this study, the original raw vibration signals are used directly as input without any pre-filtering, denoising, or feature pre-extraction, so that the phase-space reconstruction and recurrence plot construction operate on the unprocessed acquired waveforms.

Bearing faults were introduced and observed near the conclusion of the experiments. Dataset 1 includes an inner-race fault in Bearing 3 and a rolling-element defect in Bearing 4. Dataset 2 contains an outer-race fault in Bearing 1, while Dataset 3 contains an outer-race fault in Bearing 3.

**Figure 1:**
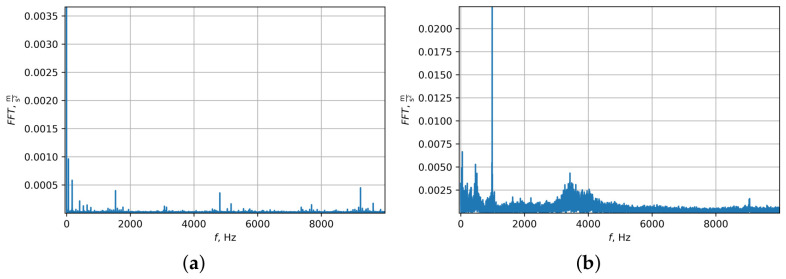
Fast Fourier Transform (FFT) spectra of vibration signals: (**a**) Channel 3 (healthy condition); (**b**) Channel 4 (defective condition). The zero-frequency component is normalized by the sample size, while all other components are scaled by half the sample size.

Figure 1 illustrates the frequency spectra of vibration signals for representative healthy and defective cases. Defective bearings have strong low-frequency components, whereas healthy bearings show more noticeable mid-frequency responses that are clearly different from white noise. This difference shows that frequency-domain representations are effective and supports the use of time-delay embedding to extract features from vibration signals.

### 2.2. The Standard Recurrence Plot

Dynamical systems often exhibit recurrences, meaning that their trajectories in phase space return close to previously visited states after some time. A recurrence plot provides a two-dimensional visualization of these recurrences by indicating, for each moment in time, when the system revisits approximately the same region of phase space as at other times. This method, introduced in [24], reveals the temporal structure of recurrences by showing which states (i.e., values of the time series) reappear after specific time delays.

The recurrence plot is represented as a binary matrix that indicates whether two states (values of the time series) are sufficiently close in phase space, according to a chosen threshold:

(1) $$R\left(i,j\right)=\left\{\begin{array}{cc} 1, & \left|x_{i}-x_{j}\right|\leq \varepsilon ,\\ 0, & \mathrm{otherwise}. \end{array}\right.$$

where *R* is a square dichotomous matrix; ${\left\{x_{k}\right\}}_{k=1}^{n}$ is the time series; ε is the threshold regulating the darkness of the recurrence plot. The recurrence plot would be almost black for an experimentally generated time series at ε=0 as the probability of the occurrence of two identical values $x_{i}$ and $x_{j}$, i≠j is very low.

The visual appearance of the dichotomous textures generated in the recurrence plot can provide some qualitative information about the dynamics represented by the time series [25].

### 2.3. Color Recurrence Plots

The concept of color recurrence plots is described in detail in [27]. The construction of the color recurrence plot exploits optimal time delays of the *d*-dimensional embedding of the given time series. Given a vector of time delays $T=\left[\tau_{1},\tau_{2},\ldots ,\tau_{d-1}\right]$; $\tau_{k}\in N$; k=1,2,…,d−1, the following subsets of composite time delays are formed:

(2) $$\left\{\tau_{1},\tau_{2},\ldots ,\tau_{d-1}\right\};\;\;\;\left\{\tau_{1}+\tau_{2},\tau_{2}+\tau_{3},\ldots ,\tau_{d-2}+\tau_{d-1}\right\},\ldots ,\;\;\;\left\{\tau_{1}+\tau_{2}+\ldots +\tau_{d-1}\right\}.$$

This grouping of time delays results in $\left(d-1\right)+\left(d-2\right)+\ldots +1=\frac{d(d-1)}{2}$ composite time delays. Next, the dimension *m* of the square color recurrence plot is defined.

Let us denote the current composite time delay as $\tau_{k}$, $k=1,2,\ldots \frac{d(d-1)}{2}$. Then, the current dichotomous digital share is defined as:

$$\begin{array}{ccc} S\left(\tau_{k},\varepsilon_{k}\right) & = & \left[\begin{array}{cccc} r(1,1+\tau_{k}) & r(2,2+\tau_{k}) & \ldots & r(m,m+\tau_{k})\\ r(m+1,m+1+\tau_{k}) & r(m+2,m+2+\tau_{k}) & \ldots & r(2m,2m+\tau_{k})\\ \vdots & \vdots & \ddots & \vdots \\ r(m^{2}-m+1,m^{2}-m+1+\tau_{k}) & r(m^{2}-m+2,m^{2}-m+2+\tau_{k}) & \ldots & r(m^{2},m^{2}+\tau_{k}) \end{array}\right], \end{array}$$

where

(3) $$r\left(i,j\right)=\left\{\begin{array}{cc} 1, & \left|x_{i}-x_{j}\right|\leq \varepsilon_{k},\\ 0, & \mathrm{otherwise}, \end{array}\right.$$

and $\varepsilon_{k}$ is individually selected for each share to produce the same number of black and white pixels.

Finally, the color recurrence plot reads [27]:

(4) $$C\left(\tau_{1},\tau_{2},\ldots ,\tau_{d-1}\right)=\sum_{k=1}^{\frac{d\left(d-1\right)}{2}}S\left(\tau_{k},\varepsilon_{k}\right),$$

The total number of different values each pixel in *C* could obtain is $\frac{d(d-1)}{2}+1$ which is referred to as the number of colors in the plot.

### 2.4. Non-Uniform Embedding

Let the embedding dimension of the time series ${\left\{x_{k}\right\}}_{k=1}^{n}$ be set as *d*. Non-uniform embedding maps the time series to a trajectory matrix:

(5) $$X_{k}=\left[x_{k},x_{k+\tau_{1}},x_{k+\tau_{1}+\tau_{2}},\ldots ,x_{k+▵}\right],\;\;\;k=1,2,\ldots ,n-▵,$$

where $\tau_{k}\in N$, k=1,2,…,d−1, and $▵=\tau_{1}+\tau_{2}+\ldots +\tau_{d-1}$.

Then, the set of optimal time delays $T=\left[\tau_{1},\tau_{2},\ldots ,\tau_{d-1}\right]$ can be determined by maximizing the following target function *F* [33]:

(6) $$F\left(\tau_{1},\tau_{2},\ldots ,\tau_{d-1}\right)=\frac{1}{\left(n-▵\right)\sqrt{d}}\sum_{k=1}^{n-▵}\sqrt{x_{k}^{2}+x_{k+\tau_{1}}^{2}+\ldots +x_{k+▵}^{2}}.$$

Prior to the computation of the target function *F*, the signal mean is subtracted from the time series, ensuring that the geometric separation of the reconstructed state vectors is not biased by a non-zero offset. By maximizing this target function, the delay vector is selected to enhance the geometric separation of the reconstructed state vectors while keeping the procedure computationally simple.

### 2.5. The Motivation for This Work

As mentioned earlier, our aim is to find the best uniform set of time delays for vibrational time series (in terms of the target function described by Equation (6). The main motivation of such an approach is a hypothesis formulated as follows:

**Hypothesis** **1.** *The goodness of the best uniform set of time delays is not much worse than that of the globally best nonuniform set of time delays.*

In order to check our heuristic approach, an exhaustive search is performed by evaluating the target function for each non-uniform time delay vector $\left[\tau_{1},\tau_{2},\ldots ,\tau_{d-1}\right]$, $\tau_{k}\in \left\{1,2,\ldots ,50\right\}$, $k\in \bar{1,d-1}$. For the embedding dimension d=5 a total of $50^{5-1}=6,250,000$ permutation computations would be needed. On the other hand, considering only uniform time delay vectors, only 50 different vector computations are required, which is much lower than in the non-uniform case: $\left\{1,1,\ldots ,1\right\}$, $\left\{2,2,\ldots ,2\right\}$, …, $\left\{50,50,\ldots ,50\right\}$.

Brute-force search for the target function of uniform delay vectors was performed for the chosen data files in each of the Dataset 3 channel. Results for the top five uniform delay vectors are shown in Table 2. CH1, CH2 and CH4 represent healthy bearings; only the first data files were considered for the search. CH3 represents a degrading bearing; only the last data file was considered, and it corresponds to zero RUL. Computations for CH2 and CH3 resulted in (49,49,49,49) being the global best uniform vector. The best value for CH1 and CH4 was computed for the delay vectors (47,48,48,47) and (22,25,21,47) respectively. The vector (49,49,49,49) is ranked 29,391 in terms of the target function and only 0.1797% worse compared to the global best vector for CH4 data.

**Figure 2:**
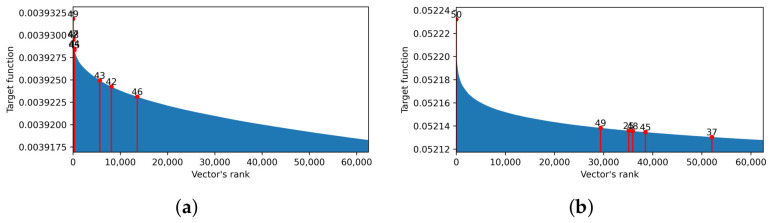
Top 1% values of the target functions of delay vectors. The red lines mark target function values for uniform delay vectors and the numbers correspond to the time delay values. Part (**a**) corresponds to CH3 (RUL is 0); part (**b**) corresponds to CH4 (no defect).

The top 1% highest values of the target function *F* are depicted in Figure 2. The values are sorted and exhibit exponential decay. The results for CH3 are particularly interesting because the best uniform delay vector is also the best global one, and the exponential decay in *F* emphasizes its prominence. Figure 2 also acts as a brute-force confirmation of the hypothesis, although only for a specific dataset.

Considering the fact that the delay vector (49,49,49,49) proved to be a good replacement for the global best vector if the target function *F* is a criterion for the choice, it is further used for experimental validation.

## 3. Results

In this section, the best uniform delay vector will be used for the construction of standard and color recurrence plots and then prediction of RUL. The accuracy of the prediction is an alternative metric to assess the quality of the best uniform delay vector.

### 3.1. Computing Color Recurrence Plots

**Figure 3:**
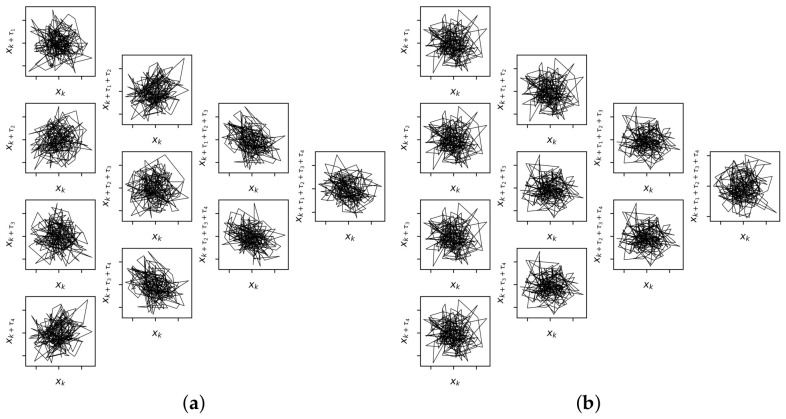
Two-dimensional phase plane projections of time-delayed vibration data (first 250 points) of CH4 (no defect). Part (**a**) corresponds to time delays from global best delay vector (22,25,21,47), part (**b**) corresponds to time delays from best uniform delay vector (49,49,49,49).

The first step in computing color recurrence plots is to perform two-dimensional time-delay embedding using all possible variants of sums of time delays. Figure 3 shows the phase plane projections of time-delayed CH4 vibration data. Coordinates of the projections are next used for the construction of standard recurrence plots. Both best global and best uniform time delay vectors yield projections that visually cover the plane in a more or less uniform way. Additionally, no visual differences of the attractors are observed. This is not unexpected since CH4 represents bearing with no defect. On the contrary, dataset description states that both defective and healthy bearings are operating on the common shaft which may suggest two remarks. One is that the vibrations of the bearings might be coupled to some degree. Another remark is on the possible need for further data processing; in this case, that is computing (color) recurrence plots.

The embedding dimension is fixed at d=5 and the upper limit for time delays is set to L=50. Each color recurrence plot is constructed as a square matrix of dimension 142×142 pixels. The number of samples from each data file used to construct a single plot is $142^{2}+\sum_{k=1}^{d-1}\tau_{k}$, which in the worst case yields $142^{2}+d\cdot L=20,164+200=20,414$ samples, safely within the 20,480 samples available per file. For the optimal uniform delay vector (49,49,49,49), the exact number of samples used is 20,164+196=20,360.

**Figure 4:**
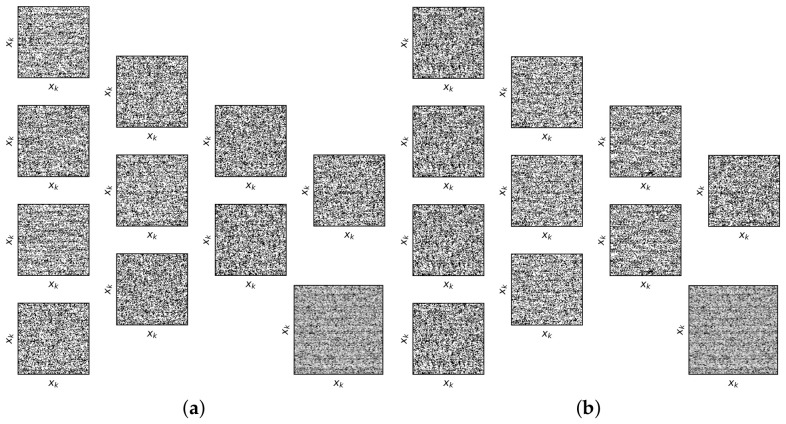
Dichotomous digital shares and corresponding color recurrence plots for CH4 data (no defect, middle of the experiment). Part (**a**) corresponds to the use of the time delays from global best delay vector (22,25,21,47); part (**b**) corresponds to using the time delays from best uniform delay vector (49,49,49,49).

Figure 4 shows respective digital shares (standard recurrence plots) for each variant of time delay sums. The average standard recurrence plot for each time delay vector is shown in the bottom right of parts (a) and (b) in Figure 4. By definition, these are color recurrence plots. It is important to note that the threshold ε for each recurrence plot representation is calibrated once at the beginning of the experiment using data from the healthy bearing, and this calibrated value is subsequently kept frozen for all remaining files throughout the run-to-failure experiment. Consequently, the progressive evolution of the recurrence plot intensity observed across the degradation trajectory reflects genuine changes in the bearing dynamics rather than any variation in the threshold parameter.

**Figure 5:**
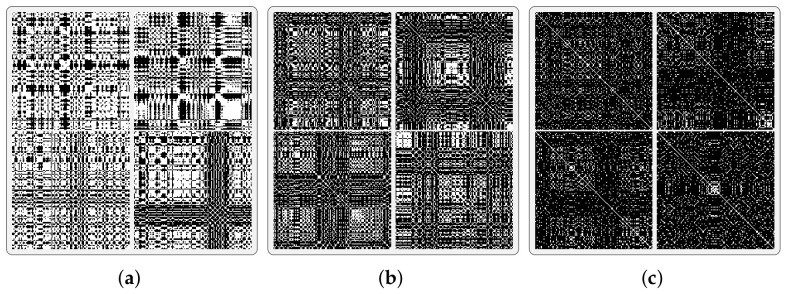
Standard recurrence plots for the vibrational signal corresponding to maximal RUL in (**a**), RUL equal to 24 h in (**b**) and minimal RUL in (**c**). Visually, the patterns do not show a consistent change in the structure throughout the experiment.

Next, recurrence plots need to be computed for each data file and in this way represent a visual feature of the vibrational signal at a given moment in time. Figure 5 shows standard recurrence plots for the vibrational signal. Two main observations could be made. First, the plots are visually different between the signals corresponding to high and low RUL. Additionally, the plots are getting darker as RUL decreases, and this is more pronounced at the end of the experiment. Secondly, one also sees that a group of images corresponding to either low or high RUL consists of images having different patterns. Visually, the patterns evolve throughout the experiment but do not show a consistent change in the structure. In general, such variety might be a problem for machine learning algorithms in achieving high accuracy regression.

**Figure 6:**
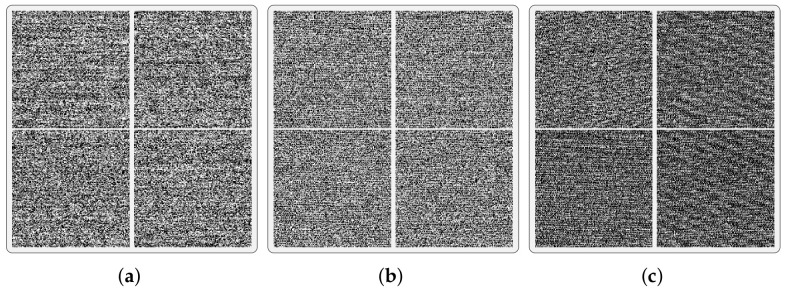
Color recurrence plots (algorithm B) for the vibrational signal corresponding to maximal RUL in (**a**), RUL equal to 24 h in (**b**) and minimal RUL in (**c**). Faint diagonal patterns are emerging when RUL is decreasing.

Now, if the color recurrence plots are considered, the insights are different. The resulting plots are shown in Figure 6. At first glance, the plots are rather similar and noisy, but looking closer, it is observed that faint diagonal patterns start to emerge at the end of the experiment. The darkening is also observed, and it is less misleading because of a more uniform scattering of shades of gray compared to the scattering of white and black pixels in standard recurrence plots. Thus, it could be expected that the prediction of RUL will be more accurate when using color recurrence plots.

It should be noted that the grayscale rendering of the color recurrence plots is a deliberate choice: since matrix *C* is used directly for numerical statistical feature extraction, the smooth and monotonically evolving global intensity is more informative for RUL prediction than a colormap applied purely for visual effect.

### 3.2. Numerical Characteristics of Recurrence Plots

A set of numerical characteristics for standard recurrence plots and for color recurrence plots are considered here as the final set of characteristics for the prediction of RUL. The characteristics used here are: average value μ, median $m_{e}$, standard deviation σ, skewness $a_{s}$, kurtosis *k* and Shannon entropy *E*. Each of them are computed using the elements of matrices *R* and *C*. In this way, a segment of vibrational data is transformed to a list of six numbers.

**Figure 7:**
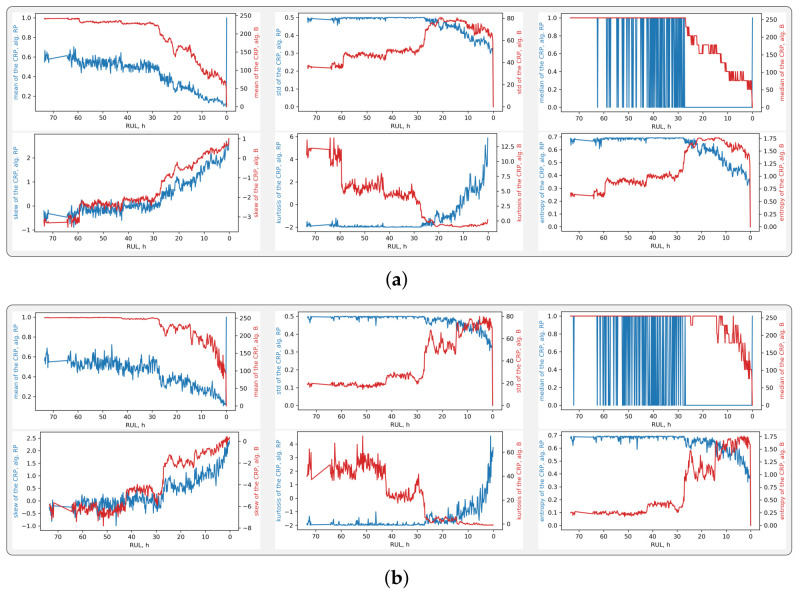
Standard statistical measures for values of patterns obtained by algorithms RP and B. Part (**a**) represents CH3 (defect is developing); part (**b**) represents CH4 (no defect). Reading in rows, the plots correspond to the average value μ, median $m_{e}$, standard deviation σ, skewness $a_{s}$, kurtosis *k* and Shannon entropy *E* respectively.

The comparison between the basic statistical measures of the recurrence patterns obtained by algorithms RP and B is shown in Figure 7. Part (a) corresponds to CH3 and part (b) to CH4; regardless, the tendencies of the numerical characteristics are visually similar. Both algorithms result in features which are changing more as RUL decreases. It is also seen that the features do not really change until about the last 24–72 h of the experiment. The same was also applicable to the recurrence patterns. This suggests that the best RUL prediction will be achieved by using only last observations, for example, last 24–72 h. If the whole vibrational data series was used, the first observations would have similar recurrence patterns and in turn similar features representing high RUL values.

### 3.3. Predicting RUL from Color Recurrence Plots

Three methods were used for RUL prediction out of the numerical characteristics mentioned above: XGBoost regressor, Random Forest Regressor (RFR) and Gradient Boosted Regressor (GBR).

CH3 data were used for the RUL prediction, and 72 h of the last observations was chosen. Training and testing subsets were randomly sampled from the chosen data with the proportion 80/20 respectively. The sampling was performed each time before the training, which was repeated for 21 individual iterations. XGBoost was optimized for training by performing 5-fold cross-validated grid search using maximum depth of 3 to 5, η=0.1 or η=0.01 and 500 estimators. This was achieved by the *GridSearchCV* function from Python’s *scikit-learn* 1.8.0 library. RFR optimization was performed by iteratively considering maximum depths from 2 to 10. Standard parameters were used for the GBR method.

The prediction errors were then averaged in the test subset and the summary is provided in Table 3. GBR was shown to be the best prediction method that offers the highest $r^{2}$ if the RP algorithm is used. The RFR showed even better accuracy for the case of algorithm B.

**Figure 8:**
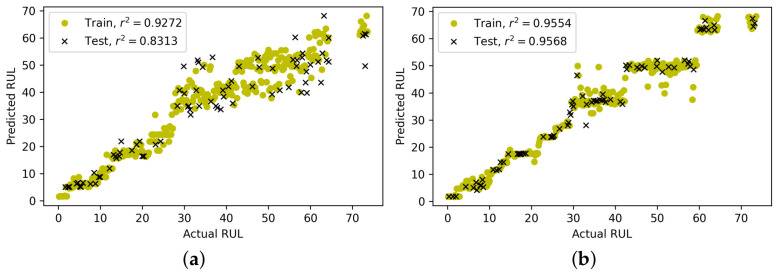
Prediction of RUL using CH3 data. Part (**a**) shows the result of GBR with algorithm RP; part (**b**) corresponds to RFR with algorithm B.

It must be noted that 400 last files in the dataset were used for the prediction. This corresponds to RUL between about 70 and 0 h. Predictions of longer values would result in output fluctuations around RUL=70. Figure 8 depicts the comparison between RUL prediction in training and testing samples. No trace of overfitting is observed; predictions for lower values of RUL have a higher accuracy overall. It could also be mentioned that the prediction of RUL may be carried out using CH1, CH2, or CH4 data. This is because all four bearings are mounted on the same shaft and defect-induced disturbances may affect vibration measurements of the healthy bearings. Such predictions were also performed, and the accuracies were of comparable magnitude.

## 4. Conclusions

This paper addressed the suitability of uniform time-delay embedding as a near-optimal substitute for globally optimized non-uniform embedding in recurrence-based bearing vibration analysis. The central hypothesis—that the best uniform delay vector performs not significantly worse than the globally best non-uniform delay vector with respect to the attractor reconstruction target function—was confirmed empirically through exhaustive brute-force search on the IMS bearing dataset. For the defective channel CH3, the best uniform delay vector (49,49,49,49) coincided with the global optimum, while for the healthy channel CH4 it ranked 29,391th among over six million candidate vectors yet remained within 0.1797% of the global best target function value. This near-optimality result has an important practical consequence: the combinatorial search over non-uniform delay coordinates—which scales as $50^{4}$ for embedding dimension five—can be safely replaced by a scalar sweep over just 50 candidate uniform delays without meaningful loss of phase-space reconstruction quality.

Building on this result, color recurrence plots were constructed from the optimal uniform embedding and applied to RUL prediction on the IMS dataset using three regression methods: XGBoost, Random Forest Regressor (RFR), and Gradient Boosted Regressor (GBR). A key finding is that standard binary recurrence plots, despite conveying rich local dynamical information, exhibit erratically varying patterns across degradation stages that impede reliable regression. Color recurrence plots, by contrast, suppress these local variations through averaging across multiple phase-space projections, yielding a globally evolving intensity that produces clear and interpretable degradation indicators far more amenable to data-driven RUL estimation. This advantage is reflected clearly in the prediction results: the best model using color recurrence plot features achieved an $r^{2}$ of 0.9348 and MAE of 3.47 h, compared to $r^{2}=0.8155$ and MAE of 5.97 h for standard recurrence plots, a substantial improvement across all metrics.

The analysis also revealed that recurrence-based features remain largely stationary throughout most of the bearing lifetime, changing meaningfully only during the final 24–72 h before complete failure. This behavior motivates a practical prognostic strategy: monitor continuously but activate RUL estimation upon detection of accelerated degradation onset, from which point color recurrence plot features provide accurate and stable predictions.

Furthermore, the averaging mechanism inherent to color recurrence plot construction provides implicit robustness to noise: by summing multiple binary dichotomous shares across all composite delay projections, noise-induced local pattern variations cancel out, leaving only the globally evolving degradation trend available for statistical feature extraction.

Taken together, these findings establish uniform delay embedding combined with color recurrence representation as an efficient, principled, and practically deployable framework for recurrence-based bearing condition monitoring. The approach reduces embedding parameter complexity without sacrificing prognostic utility, making it well-suited for real-time predictive maintenance in industrial environments.

A limitation of the proposed framework is that it is optimally suited for the accelerated degradation phase; the detection of incipient weak faults, where fault signatures are submerged in strong noise, lies outside the scope of the present study and remains an important direction for future research.

Further research in addressing the validation protocol by adopting a chronological train/test split would provide a more conservative and temporally realistic assessment of the predictive performance of the proposed framework. Additionally, extending the near-optimality validation to other bearing datasets and varying operating conditions represents a possible direction for future research.

A systematic comparison of the proposed framework with end-to-end deep learning approaches, such as convolutional neural networks and deep temporal models, is also identified as a possible further research path.

## Figures and Tables

**Table 1:** Overview of the IMS experimental dataset.

Attribute	Dataset 1	Dataset 2	Dataset 3
No. of Files	2156	984	4448
No. of Channels	8	4	4
Bearings	1 (CH1&2)	1 (CH1)	1 (CH1)
	2 (CH3&4)	2 (CH2)	2 (CH2)
	3 (CH5&6)	3 (CH3)	3 (CH3)
	4 (CH7&8)	4 (CH4)	4 (CH4)

**Table 2:** Top 5 uniform delay vectors with respect to the target function. $F_{★}$ is the global best target function of all time delay vectors.

CH1 (No Defect, $F_{★}=0.0746$)			CH2 (No Defect, $F_{★}=0.0908$)		
Rank *r*	$\pi_{r}$	$\frac{F_{★}-F_{\pi }}{F_{★}}\cdot 100\%$	Rank *r*	$\pi_{r}$	$\frac{F_{★}-F_{\pi }}{F_{★}}\cdot 100\%$
5	$\left(48,48,48,48\right)$	0.000226	1	$\left(49,49,49,49\right)$	0
73	$\left(29,29,29,29\right)$	0.000543	51	$\left(47,47,47,47\right)$	0.026994
2639	$\left(47,47,47,47\right)$	0.001208	298	$\left(48,48,48,48\right)$	0.050207
3370	$\left(46,46,46,46\right)$	0.001255	2387	$\left(34,34,34,34\right)$	0.085266
5610	$\left(24,24,24,24\right)$	0.001351	4794	$\left(35,35,35,35\right)$	0.100655
**CH3 (RUL Is 0, $F_{★}=0.0039$)**			**CH4 (No Defect, $F_{★}=0.0522$)**		
Rank *r*	$\pi_{r}$	$\frac{F_{★}-F_{\pi }}{F_{★}}\cdot 100\%$	Rank *r*	$\pi_{r}$	$\frac{F_{★}-F_{\pi }}{F_{★}}\cdot 100\%$
1	$\left(49,49,49,49\right)$	0	1	$\left(50,50,50,50\right)$	0
44	$\left(47,47,47,47\right)$	0.056034	29,391	$\left(49,49,49,49\right)$	0.179618
61	$\left(48,48,48,48\right)$	0.059502	35,097	$\left(25,25,25,25\right)$	0.184219
237	$\left(44,44,44,44\right)$	0.084487	35,915	$\left(48,48,48,48\right)$	0.184840
284	$\left(45,45,45,45\right)$	0.088271	38,506	$\left(45,45,45,45\right)$	0.186641

**Table 3:** Comparison between average RUL prediction errors using standard recurrence plots (RPs) and recurrence plots with algorithm B as features for CH3 data. Predictions were performed 21 times and then average values of MAE, RMSE and $r^{2}$ were computed. The best prediction errors and the highest $r^{2}$ are shown in bold.

Algorithm	Prediction Method	MAE	RMSE	*r* ^2^
RP	XGBoost	8.4379	10.2706	0.7181
	RFR	6.6309	9.3110	0.7636
	GBR	**5.9736**	**8.3340**	**0.8155**
B	XGBoost	7.2035	8.5887	0.8096
	RFR	**3.4689**	**4.8139**	**0.9348**
	GBR	3.6558	5.2350	0.9242

## Data Availability

The original data presented in the study are openly available in the NASA Prognostics Data Repository [3] at https://phm-datasets.s3.amazonaws.com/NASA/4.+Bearings.zip, accessed on 5 May 2026.
